# Human Monkeypox: Old virus with new Epidemiological and Transmission Trends

**DOI:** 10.12669/pjms.38.8.6978

**Published:** 2022

**Authors:** Sultan Ayoub Meo

**Keywords:** Human Monkeypox

## Abstract

Human monkeypox is an emerging zoonotic disease caused by a monkeypox virus. The monkeypox virus history originated in 1958 after the occurrence of a pox-like illness in monkeys. In September 1970, the first case of human monkeypox was identified in the Democratic Republic of Congo, Africa. This year, from January 01, to September 30, 2022, the virus swiftly spread from endemic to non-endemic counties, involving 106 states, infecting 68,017 people; 689 cases from 07 endemic African countries and 67,328 cases in 99 non-endemic countries in Europe, America, Asia and Oceania continents. The disease caused 27 deaths in 13 countries worldwide. The human monkeypox disease significantly affects the population in South, North and Central America 34767 (51.11%), Europe 32047 (47.11%), Africa 707 (1.03%), Asia 351 (0.51%), and Australia and Oceania continent 145 (0.21%). The occurrence of the disease is high in males with age ranges of 21-55 years. The common clinical features in monkeypox patients are skin rashes (95%), fever (72%), malaise (69%), chills (67%), pruritis (64%), headache (64%), enlarged lymph nodes (63%) myalgia (60%) and nausea and vomiting (20%). The transmission trends of the disease are rapidly changing; the virus is not limited to close contact with humans. It can spread through body fluids, respiratory droplets, and sexual contact. The disease can transmit during travelling, contact with soiled materials, infected cloths, bed linen, objects, air pollutants, and in various workplace environments. The monkeypox virus has adopted multiple transmission routes, and swiftly spreading and developing challenging and threatening situations worldwide.

## INTRODUCTION

Human monkeypox disease is caused by a monkeypox virus (MPXV), which is a zoonotic infection commonly found in African countries.^1^ The MPXV belongs to the “genus *Orthopoxvirus*, subfamily *Chordopoxvirinae* and family *poxviridae*”. The genomes of these family viruses are ≈200 kb long, with a dominant replication part and genetic material. The monkeypox family viruses are consisting of numerous pathogens and infect humans.^2^,^3^ Initially, the disease was restricted to the endemic regions of Central and Western African countries. This year from May 2022, the virus swiftly spreading and developing a highly challenging and threatening situation worldwide.^4^ The monkeypox outburst was declared a global public health emergency.

### Old virus with new history:

The monkeypox virus was first found in 1958 after the occurrence of a pox-like ailment in monkeys, which were housed in a research institute in Denmark, hence the disease became popular with the monkeypox name.^5^ About 12 years later, in 1970, the MPXV was first time identified in humans, when a nine months old infant with smallpox-like clinical features was admitted to the hospital in the Democratic Republic of Congo.^6^-^8^

In the same year, in October 1970 and May 1971, six cases of human MPXV were identified in “Liberia, Nigeria, and Sierra Leone”.^8^ The first case of MPXV was found in Nigeria in 1971, and 10 cases were reported between the years 1971 and 1978.^9^ After that, thousands of cases were confirmed in different African countries including “Benin, Cameroon, Central African Republic, the Democratic Republic of the Congo, Gabon, Cote d’Ivoire, Liberia, Nigeria, Republic of the Congo, Sierra Leone, and South Sudan”, where the MPXV is endemic.^3^

In the new millennium, the year 2003, the human monkeypox disease was first time spread from endemic African regions to non-endemic nations, including the United States of America (USA).^10^ This was the first incidence that human monkeypox was spread and identified outside of Africa and found in the USA^10^ In 2003, 47 confirmed and suspected cases of monkeypox infection were reported from six states in America. These states include Indiana, Kansas, Illinois, Ohio, Wisconsin and Missouri. The people who suffered from monkeypox infection had a history of contact with pet prairie dogs, housed near the small mammals imported from Ghana, which led to the occurrence of the first case of human monkeypox infection outside Africa.^11^,^12^ The outbreak of monkeypox in the USA^10^ reflects that virus has the capability of dispersal to animal reservoirs outside central African regions.^12^

### Monkeypox with new transmission trends:

The possible route of transmission of MPXV is “animal-human and human-human”. The respiratory droplets, direct or indirect contact with body fluids, pieces of stuff, skin lesion of an infected person^1^, and contaminated patient’s environment have been associated with inter-human transmission.^13^ The spread of the disease can occur due to physical contact and exposure to body secretions and excretions of infected individuals and contaminated materials.^1^,^13^ The number of monkeypox cases and the geographical spreading range of the disease have expanded. Since May 2022, the rapid occurrence of the disease in various nations has been unusual, which raises international concerns about a possible change in the pattern of spread of the monkeypox disease that poses a global threat.^4^

The monkeypox virus DNA lies on the surfaces in hospitals and households, and is found on bed linen, air and dust samples in rooms used by monkeypox patients.^13^ Atkinson et al., 2022^14^ reported that the MPXV was found in a workplace environment occupied by an individual with monkeypox illness.^14^ These facts demonstrate that the transmission trends of the monkeypox virus changed from close contact to other routes including body fluids, respiratory secretions, and polluted personal objects which can contaminate the environment^13^,^14^, and result in the occurrence of disease worldwide.

### Travel allied spread of the disease:

This year, from May 2022, monkeypox diseases globally involved 106 countries.^15^ The virus transmits through the infected individuals, contaminated materials, polluted personal objects, and contaminated environment resulting in the virus spreading among the people.^13^,^14^

In Nigeria, during 2017-2018, the outbreak involved 118 cases, and from September 2018 -May 2021, six people with a travelling history from Nigeria were diagnosed with monkeypox. The four people travelled to the “United Kingdom, one to Singapore and one to Israel. In July 2021, a man travelled from Nigeria to Dallas, Texas was diagnosed with monkeypox. Among 194 monitored contacts, 144 had a history of flight contacts.^16^

In the Republic of Korea, in June 2022, a monkeypox case has been reported in a person with a travel history to Europe, day after his return to the Republic of Korea, the patient was diagnosed with monkeypox.^17^ On June 24, 2022, the first confirmed case of monkeypox virus in a 20-year-old young has been reported in Taiwan, the patient returned from Germany.^18^ These facts suggest that the monkeypox virus can spread in subjects who have recent travel history, which shows that travel is a risk factor to spread the monkeypox disease.

### Global epidemiological trends:

This year, from January 2022, to September 30, 2022, the virus has adopted multiple transmission paths and swiftly spread from endemic to non-endemic counties, involving 106 states, infecting 68,017 people; 689 cases from 07 endemic African countries and 67,328 cases in 99 non-endemic countries in Europe, Central, South and North America, Australia, and Asia^19^ (Table-I). The disease caused 27 deaths in 13 countries worldwide.^19^

**Table-I:** Global distribution of human monkeypox cases.

Country	Cases
***European Countries- Non-endemic countries***
Spain	7149
France	3999
United Kingdom	3635
Germany	3621
Netherlands	1223
Portugal	917
Italy	846
Belgium	770
Switzerland	513
Austria	310
Sweden	192
Poland	188
Denmark	185
Ireland	183
***Other non-endemic countries (USA, Australia, Middle East)***
United States of America	25612
Brazil	7624
Peru	2449
Colombia	2042
Mexico	1627
Canada	1396
Chile	880
Argentina	326
Israel	252
Bolivia	185
Australia	136
Bolivia	185
Ecuador	120
***iii. Endemic countries in Africa***
The Democratic Republic of the Congo	174
Nigeria	400
**iv. Other countries**
Other countries	878
Total cases	68017

***Note:*** Cases are reported from Jan 1, 2022, to September 30, 2022.^19^

**Fig.1:**
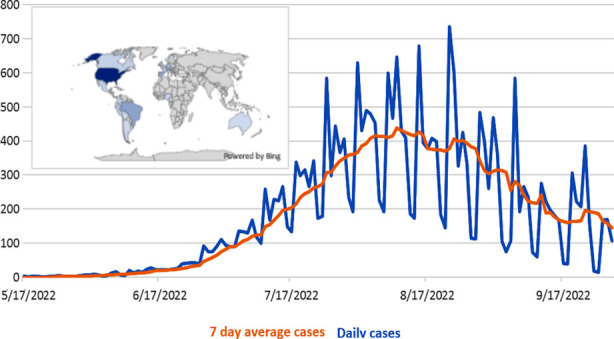
World map shows the global prevalence of human monkeypox cases, and a line graph shows transmission trends of monkeypox cases in the USA.

The human monkeypox disease significantly affected the population in European countries and the United States of America (Table-I, Fig.1). The number of cases in European countries is 32047 (47.11%), South, North and Central America 34767 (51.11%), Africa 707 (1.03%), Asia 351 (0.51%), and Australia and Oceania continent 145 (0.21%).

In Europe, the maximum number of cases reported from Spain is 7149, Germany 3621, United Kingdom 3635, France 3999, Netherlands 1223, Portugal 917, Italy 846, Belgium 770, Switzerland 513, and Austria 310.^19^ In the United States, the total number of monkeypox cases is 25612 (Table-I). The disease involved major states including New York 3914; California 4886, Florida 2520, Texas 2311, Georgia 1785, Illinois 1306, Pennsylvania 758, New Jersey 706, Maryland 657, Washington 600, Columbia 497, Virginia 464, and Massachusetts 396.^19^ The disease also swiftly spread in Canada with 1396 cases.^17^ In the USA, the incidence of monkeypox cases markedly increased in August 2022 (Fig.1). The monkeypox disease is common in males in the age range 21-55 years.^15^ These facts demonstrate that the monkeypox virus has adopted multiple transmission routes, hence swiftly spreading and developing challenging and threatening situations worldwide.^6^ The common clinical symptoms among monkeypox patients are skin rashes (95%), fever (72%), malaise (69%), chills (67%), pruritis (64%), headache (64%), enlarged lymph nodes (63%) and myalgia (60%).^15^

## CONCLUSIONS

The monkeypox virus history originated in 1958, and the first case of human monkeypox was identified in the Democratic Republic of Congo, Africa in September 1970. The virus has adopted multiple transmission paths and swiftly spreading from endemic to non-endemic countries. This year, from January 01, to September 30, 2022, the virus involved 106 countries, infecting 68,017 people; 689 cases from 07 endemic African countries, and 67,328 cases in 99 non-endemic countries worldwide. The human monkeypox disease sternly involved the entire European countries and the United States of America. The transmission trends of human monkeypox disease have rapidly changed, and the virus is not limited to close contact with humans. It can spread through body fluids, respiratory droplets, and sexual contact. The virus can spread through travel, contact with soiled materials, infected cloths, bed linen, objects, and through air pollutants and exposure to various workplace environments. The global health authorities must implement strict policies to control the burden of the disease both at regional and international levels. As the world has still not completely recovered from the major shake-up of COVID-19, society cannot afford another pandemic of human monkeypox.
